# Advanced *in Vitro* Safety Assessment of Herbal Medicines for the Treatment of Non-Psychotic Mental Disorders in Pregnancy

**DOI:** 10.3389/fphar.2022.882997

**Published:** 2022-06-23

**Authors:** Deborah Spiess, Moritz Winker, Alexandra Dolder Behna, Carsten Gründemann, Ana Paula Simões-Wüst

**Affiliations:** ^1^ Department of Obstetrics, University Hospital Zurich, University of Zurich, Zurich, Switzerland; ^2^ Center for Complementary Medicine, Faculty of Medicine, University of Freiburg, Freiburg, Germany; ^3^ Translational Complementary Medicine, Department of Pharmaceutical Sciences, University of Basel, Basel, Switzerland

**Keywords:** mental health disorders, pregnancy, safety, *Hypericum perforatum*, *Eschscholzia californica*, *Valeriana officinalis*, *Lavandula angustifolia*, *Humulus lupulus*

## Abstract

When confronted with non-psychotic mental disorders, pregnant women often refrain from using synthetic drugs and resort to herbal medicines such as St. John’s wort, California poppy, valerian, lavender, and hops. Nevertheless, these herbal medicines have not yet been officially approved in pregnancy due to lack of safety data. Using a variety of *in vitro* methods (determination of cytotoxicity, apoptosis induction, genotoxicity, effects on metabolic properties, and inhibition/induction of differentiation) in a commonly used placental cell line (BeWo b30), we were previously able to show that extracts from these plants are likely to be safe at the usual clinical doses. In the present work, we wanted to extend our safety assessment of these herbal medicines by 1) looking for possible effects on gene expression and 2) using the same *in vitro* methods to characterize effects of selected phytochemicals that might conceivably lead to safety issues. Proteomics results were promising, as none of the five extracts significantly affected protein expression by up- or down-regulation. Protopine (contained in California poppy), valerenic acid (in valerian), and linalool (in lavender) were inconspicuous in all experiments and showed no adverse effects. Hyperforin and hypericin (two constituents of St. John’s wort) and valtrate (typical for valerian) were the most obvious phytochemicals with respect to cytotoxic and apoptotic effects. A decrease in cell viability was evident with hypericin (≥1 µM) and valtrate (≥10 µM), whereas hyperforin (≥3 µM), hypericin (30 µM) and valtrate (≥10 µM) induced cell apoptosis. None of the tested phytochemicals resulted in genotoxic effects at concentrations of 0.1 and 1 µM and thus are not DNA damaging. No decrease in glucose consumption or lactate production was observed under the influence of the phytochemicals, except for valtrate (at all concentrations). No compound affected cell differentiation, except for hyperforin (≥1 µM), which had an inhibitory effect. This study suggests that extracts from St. John’s wort, California poppy, valerian, lavender, and hops are likely to be safe during pregnancy. High plasma concentrations of some relevant compounds—hyperforin and hypericin from St. John’s wort and valtrate from valerian—deserve special attention, however.

## 1 Introduction

Non-psychotic mental disorders (NMDs) are a common problem during pregnancy. One multinational study found that on average 28.9% of pregnant women used herbal medicines during pregnancy, although there were significant differences between regions/countries, and that the prevalence in Switzerland was thus very high at 40.6% (Kennedy et al., 2013). In a recent survey we conducted in Switzerland, 51.3% of participants reported a mental symptom during pregnancy (such as insomnia, anxiety, and depressive mood). In addition, 13.2% and 4.0% suffered from acute and chronic mental disorders, respectively. Interestingly, only a few participants used antidepressants (e.g., sertraline, citalopram) and none mentioned (or at least indicated) the use of sedatives/anxiolytics (e.g., lorazepam, diazepam) during pregnancy. This suggests that pregnant women refrain from using synthetic psychoactive medication and prefer resorting to herbal medicines for mild NMD treatment (Gantner et al., 2021). Such behavior is well justified, as psychotropic drugs can lead to side effects for both the mother and the fetus or newborn (Howard et al., 2014; Stewart and Vigod, 2020).

Herbal candidates for the treatment of NMDs in pregnancy include St. John’s wort (*Hypericum perforatum* L.), California poppy (*Eschscholzia californica* Cham.), valerian (*Valeriana officinalis* L.), hops (*Humulus lupulus* L.), and lavender (*Lavandula angustifolia* Mill.). St. John’s wort is a herbal alternative to synthetic antidepressants in the treatment of mild to moderate depression (Wurglics and Schubert-Zsilavecz, 2006), which does not differ in efficacy from selective serotonin reuptake inhibitors (Cui and Zheng, 2016). California poppy has a long tradition of use in aiding sleep and relieving mild symptoms of mental stress (European Medicines Agency, 2015a). There are currently a variety of California poppy products on the market that are approved as phytomedicines (contain either plant extract or powdered drug), but also various dietary supplements (European Medicines Agency, 2015b; Medicinal product information, 2022). To avoid resorting to synthetic medications for sleep disorders, a combination of valerian and hops offers an alternative treatment. Several randomized trials conclude that the valerian-hops combination mentioned has (modest) hypnotic effects and significantly reduces sleep latency (Morin et al., 2005; Koetter et al., 2007; Dimpfel and Suter, 2008). Constituents of both extracts complement each other: some compounds of valerian act like endogenous adenosine and increase sleepiness, while a few phytochemicals of hops act like endogenous melatonin and support rhythmicity (Brattström, 2007). Although there are a variety of registered valerian-hops preparations on the Swiss market, there are valerian—but not hops—monopreparations (Medicinal product information search platform, 2022). Lavender essential oil is best known for its calming, sedative and anxiolytic effects and is therefore popular in the treatment of restlessness, sleep disorders, and anxiety (Schilcher et al., 2016). The efficacy and safety of a patented essential oil produced from *Lavandula angustifolia* flowers for oral administration has been demonstrated in adult patients with generalized anxiety disorder in several trials (Woelk and Schläfke, 2010; Kasper et al., 2014).

The evidence for the safety of the herbal medicines mentioned above in pregnancy is, on the other hand, very limited or non-existent. The Committee on Herbal Medicinal Products of the European Medicine Agency does not recommend the use of these herbal medicines during pregnancy due to insufficient safety data (European Medicines Agency, 2009; European Medicines Agency, 2012; European Medicines Agency, 2014; European Medicines Agency, 2015a; European Medicines Agency, 2016). In our survey, 3.5% of pregnant women reported taking St. John’s wort, 5.8% used valerian, lavender was used by 16.2% of women, and hops by 2.3% (Gantner et al., 2021).

In examining the phytochemical composition of the selected herbal candidates for treatments in pregnancy, several compounds stand out that are highly biologically active, found in various species of interest, and/or very abundant and thus might potentially cause problems from a safety perspective. In our opinion, these include hyperforin and hypericin, which are the most abundant representatives of phloroglucinol derivatives (0.2–0.4% of total dry weight of the herb) and naphthodianthrones (0.1–0.3% of total dry weight of the herb) in St. John’s wort (Blaschek, 2016). Also, protopine deserves particular attention as one of the major isoquinoline alkaloids of California poppy (Tomè et al., 1999; Fedurco et al., 2015), which is also present in several other herbal medicines (Guinaudeau and Shamma, 1982). Furthermore, we chose valerenic acid and valtrate, which are important representatives of the sesquiterpenes and the valepotriates present in valerian root (Blaschek, 2016). Finally, linalool was considered, since it corresponds to approximately one quarter (26.1%) of lavender oil and is often one of the major components in essential oils produced by a variety of plants such as hops, coriander, and star anise (Karabín et al., 2016; Schilcher et al., 2016). Linalool was identified as the major pharmacologically active constituent involved in the anti-anxiety effect of lavender essential oil (Umezu et al., 2006).

In our previous work, we conducted an initial *in vitro* assessment of the safety profile of extracts from St. John´s wort, California poppy, valerian, lavender, and hops. In concentrations up to 30 μg/ml, they did not possess any cytotoxic or genotoxic potential and did not compromise human placental cell viability, metabolic activity or differentiation (Spiess et al., 2021). In a next step, our research goals were to 1) look for possible effects of whole extracts on gene expression and 2) use the same variety of methods as before to characterize effects of particularly relevant phytochemicals from these extracts. For the above-mentioned reasons, the latter include hypericin, hyperforin, protopine, valerenic acid, valtrate, and linalool.

## 2 Materials and Methods

### 2.1 Cell Culture

The BeWo b30 cell line was provided by Dr. Tina Buerki-Thurnherr (Empa - Swiss Federal Laboratories for Materials Science and Technology, St. Gallen, Switzerland), with permission from Dr. Alan L. Schwartz (Washington University School of Medicine, MO, United States). As described in a previous study (Spiess et al., 2021), cells were cultivated in F-12K Nut Mix supplemented with 10% heat inactivated FBS, antibiotics (100 U/ml penicillin, 100 μg/ml streptavidin) and 2 mM L-glutamine (all from Gibco), at 37°C and in 5% CO_2_ humidified atmosphere.

### 2.2 Cell Treatments

All plant material was of Ph. Eur. grade. *V. officinalis* roots, *H. lupulus* flowers and *H. perforatum* herb were purchased from Dixa (lot numbers 180084, 191241, 192140, respectively). *E. californica* herb was obtained from Galke (lot number 811502). Voucher specimens (numbers 1029, 1167, 1166 and 1234, respectively) have been deposited at the Division of Pharmaceutical Biology, University of Basel. The powdered plant material was extracted with 70% EtOH by pressurized liquid extraction in a Dionex ASE 200 Accelerated Solvent Extractor. Three cycles of extraction of 5 min each were performed at a temperature of 70°C and a pressure of 120 bar. *L. angustifolia* essential oil Ph. Eur. was purchased from Hänseler (lot number 2018.01.0274). See Supporting Information of our previous work (Spiess et al., 2021) for characterization of all hydroalcoholic extracts and of *L. angustifolia* essential oil. The following phytochemicals were obtained from commercial sources: hyperforin, hyperforin dicyclohexylammonium salt and linalool (Sigma-Aldrich), protopine and valerenic acid (Extrasynthese), and valtrate (Toronto Research Chemicals). Dry herbal extracts and pure phytochemicals were dissolved in sterile DMSO (Sigma-Aldrich) and stored at -80°C. BeWo b30 cells were treated with increasing concentrations of phytochemicals (up to 30 μM, a concentration higher than all reported achieved plasma concentrations; compare with discussion) in culture medium. Control cells were exposed to a final concentration of ≤0.3% DMSO so as not to decrease cell viability. The biochemical and morphological differentiation of BeWo b30 cells was stimulated with 5 µM forskolin (FSK; Lucerna-Chem; dilution from a 30 mM stock solution prepared in DMSO). Due to light sensitivity and to avoid phototoxicity under treatment with St. John’s wort extract, hyperforin and hypericin, the relevant experiments were performed with as little light as possible.

### 2.3 Protein Analysis

#### 2.3.1 Sample Preparation

BeWo b30 cells were seeded into transparent T25 culture flasks at a density of 776′400 cells/6,000 µl/flask. After overnight incubation, they were exposed to 30 μg/ml of plant extract (*H. perforatum*, *E. californica*, *V. officinalis*, *L. angustifolia* essential oil, *H. lupulus*) or untreated control (0.06% DMSO) for 48 h. Cell culture supernatants were then discarded and attached cells were rinsed with PBS and then detached by adding a 0.25% trypsin-EDTA solution, following 1–2 min incubation (37°C). The trypsinization process was stopped by the addition of cell culture growth medium and the flasks were rinsed carefully. The cell suspensions were centrifuged (at 1,300 rpm at 23°C for 5 min). The cell pellets were washed a total of three times (by aspirating the supernatant and resuspending the cell pellet in 10 ml PBS). The cells were then counted and 1′000′000 cells per condition were centrifuged. The corresponding cell pellets were immediately snap-frozen in liquid nitrogen (without buffer) and stored at −80°C for subsequent analysis of proteins.

For each sample, proteins were extracted using a tissue homogenizer (TissueLyser II, QUIAGEN) and digested by using a commercial iST Kit (PreOmics). Briefly, 150 µl of ‘Lyse’ buffer and around 150 mg of glass beads (425–500 μm, Sigma) were added to each cell pellet. After 2 cycles of protein extraction (2 min each, 30 Hz) and incubation for 10 min at 95°C, the solubility of the extracted proteins was enhanced by processing the samples with High Intensity Focused Ultrasound for 1 min, setting the ultrasonic amplitude to 85% followed by additional incubation for 10 min at 95°C. After centrifugation for 10 min at 14′000 g the supernatant was used for further processing. The proteins (50 µg in each case) were digested on the membrane by adding 50 µl of the ‘Digest’ solution. After 60 min of incubation at 37°C the digestion was stopped with 100 µl of, Stop, solution. The samples were then centrifuged, and the supernatant was transferred to the cartridge. The solutions in the cartridge were removed by centrifugation at 3,800 *g*, while the peptides were retained by the iST-filter. Finally, the peptides were washed, eluted, dried and re-solubilized in 20 µl of injection buffer (3% acetonitrile, 0.1% formic acid). Prior to LC-MS analysis, the peptide concentrations were estimated by means of a NanoDrop spectrophotometer, and the injection amounts normalized to an absorbance of 0.3 at 280 nm.

#### 2.3.2 Liquid Chromatography-Mass Spectrometry Analysis

Mass spectrometry analysis was performed on an Orbitrap Fusion Lumos (Thermo Scientific) equipped with a Digital PicoView source (New Objective) and coupled to an M-Class UPLC (Waters). Solvent composition at the two channels was 0.1% formic acid for channel A and 0.1% formic acid, 99.9% acetonitrile for channel B. For each sample, 1 μl of diluted peptides were loaded on a commercial MZ Symmetry C18 Trap Column (100Å, 5 μm, 180 μm × 20 mm, Waters) followed by a nanoEase MZ C18 HSS T3 Column (100 Å, 1.8 µm, 75 μm × 250 mm, Waters). The peptides were eluted at a flow rate of 300 L/min by a gradient from 5 to 22% B in 77 min, 32% B in 10 min and 95% B for 10 min. Samples were acquired in a randomized order. The mass spectrometer was operated in data-dependent mode, acquiring a full-scan MS spectra (300–1,500 *m/z*) at a resolution of 120′000 at 200 *m/z* after accumulation to a target value of 500′000. Data-dependent MS/MS were recorded in the linear ion trap using quadrupole isolation with a window of 0.8 Da and HCD fragmentation with 35% fragmentation energy. The ion trap was operated in rapid scan mode with a target value of 10′000 and a maximum injection time of 50 ms. Only precursors with an intensity above 5′000 were selected for MS/MS and the maximum cycle time was set to 3 s. Charge state screening was enabled. Singly, unassigned and charge states higher than seven were rejected. Precursor masses previously selected for MS/MS measurement were excluded from further selection for 20 s, and the exclusion window was set at 10 ppm. The samples were acquired using internal lock mass calibration on *m/z* 371.1012 and 445.1200. The mass spectrometry proteomics data were handled using the local laboratory information management system (Türker et al., 2010) and all relevant data have been deposited to the ProteomeXchange Consortium via the PRIDE (Perez-Riverol et al., 2022) partner repository with the dataset identifier PXD031765.

#### 2.3.3 Protein Identification and Label Free Protein Quantification

The acquired raw MS data were processed by MaxQuant (version 1.6.2.3), followed by protein identification using the integrated Andromeda search engine (Cox and Mann, 2008). One separate MaxQuant experiment was set up for each set of sample (B015, B020, B035, and HMEV21). Spectra were searched against a swissprot canonical *Homo sapiens* proteome (taxonomy 9,606, version from 2019 to 07-09), concatenated to its reversed decoyed fasta database and common protein contaminants. Carbamidomethylation of cysteine was set as fixed modification, while methionine oxidation and N-terminal protein acetylation were set as variable. Enzyme specificity was set to trypsin/P allowing a minimal peptide length of 7 amino acids and a maximum of two missed-cleavages. MaxQuant Orbitrap default search settings were used. The maximum false discovery rate was set to 0.01 for peptides and 0.05 for proteins. Label free quantification was enabled and a 2 min window for match between runs was applied. In the MaxQuant experimental design template, each file is kept separate in the experimental design to obtain individual quantitative values. The MaxQuant results were loaded into Scaffold (Proteome Software Inc.) to validate the peptide and protein identifications. Only proteins identified with at least 2 peptides were considered for follow up analysis. The statistical significance threshold was set to an adjusted *p*-value ≤ 0.2, and a log_2_FC > ±1.

### 2.4 Functional Assays

The same plethora of functional assays was used as in our previous work with herbal extracts (Spiess et al., 2021), as briefly described in the following.

#### 2.4.1 Viability Assay

The *in vitro* cytotoxicity of the different concentrations of phytochemicals (0.01, 0.03, 0.1, 0.3, 1, 3, 10, and 30 µM) was tested using a WST-1 viability assay. BeWo b30 cells were seeded in 96-well flat-bottom plates with a density of 2 × 10^4^ cells/100 µl/well on the day before exposure to the phytochemical dilutions in fresh culture medium. Camptothecin (CPT, 300 μM; apoptosis control; Tocris Bioscience) or 0.5% Triton-X-100 (TX, necrosis control; Sigma-Aldrich) served as positive controls. After 72 h of incubation, culture supernatant was aspirated and replaced by medium without phenol red, and 5 µl Cell Proliferation Reagent WST-1 (Roche) was added. A spectrophotometric measurement was taken (450 nm) after 75 min of incubation, using a plate reader (Tecan Reader Infinite M 200).

#### 2.4.2 Apoptosis Assay

BeWo b30 cells were subjected to the same treatment described for the WST-1 assay to assess the level of apoptosis after application of the test substances. After 72 h of incubation, the cells were washed with PBS and detached using Accutase (Sigma-Aldrich). All liquids were pooled and centrifuged for 5 min (300 g), followed by an AnnexinV-FITC (eBioscience) staining, which was performed according to the manufacturer’s instructions. A flow cytometric readout was obtained and analyzed using appropriate software (BD FACScalibur, BD Biosciences, FlowJo Software).

#### 2.4.3 Comet Assay

A comet assay was used to examine the genotoxic potential of the selected phytochemicals. Microscopic slides were coated with 1% normal-melting agarose in PBS (SERVA Electrophoresis GmbH) beforehand. Cells were seeded with a density of 4 × 10^5^ cells/100 µl/well on the day before exposure. Different concentrations of phytochemicals (0.1, 1, and 10 µM) or 3 mM ethyl methanesulfonate (EMS, positive control; Sigma-Aldrich) were added for 3 h. A slightly boiling 0.7% normal-melting agarose solution (200 µl) was then applied to the precoated slides. The cells were once washed with PBS, dissolved using Accutase (Sigma-Aldrich) and resuspended in 30 µl complete medium. The cells were then gently mixed with 90 µl 0.7% low-melting agarose (SERVA Electrophoresis GmbH) solution (rapidly heated to 100°C and kept at 38°C prior to use) and added as a final layer to the slides. Electrophoresis was performed at 25 V/300 mA for 20 min. The slides were finally washed with ddH_2_O and PBS and fixed with 99% EtOH. Fixed samples were stained with ethidium bromide solution (5 μg/ml; Carl Roth GmbH), and images were taken for later analysis with CometScore software (version 2.0.038 for Windows; TriTek Corp., United States).

#### 2.4.4 Glucose and Lactate Concentration Measurements

Cells were seeded into transparent 24-well plates at a density of 2.5 × 10^4^ cells/1,000 µl/well. After overnight incubation, they were exposed to different concentrations (1, 3, 10, and 30 µM) of phytochemicals or untreated control (0.2% DMSO) for 48 h. Cell culture supernatants and pellets were collected and immediately frozen at −80°C for subsequent analysis of the metabolic parameters (glucose/lactate) and protein concentrations, respectively. Glucose and lactate were determined using an automated blood gas analyzer (ABL800 Flex, Radiometer Medical ApS). Protein concentrations were determined using the bicinchoninic acid protein assay kit (Pierce) with BSA (Thermo Scientific) as reference standard.

#### 2.4.5 Placental Cell Differentiation and β-hCG Production

To induce differentiation, cells were seeded into 24-well plates (2.5 × 10^4^ cells/1,000 µl/well) on the day before exposure to different concentrations (1, 3, 10 and 30 µM) of phytochemicals, control (0.2% DMSO) or FSK control (5 µM) for 48 h. To test the ability to inhibit differentiation, cells were seeded into 96-well flat-bottom plates (1 × 10^4^ cells/100 µl/well). After an overnight incubation, the cells were incubated with different concentrations of phytochemicals (serial dilution below apoptotic concentrations based on preliminary results), control (0.2% DMSO) or FSK control (5 µM) for 24 h. Afterwards all cells (except control) were differentiated with FSK (5 µM) for another 24 h. Analysis of *β*-hCG concentrations—a marker of placenta cell differentiation—was performed by standard ELISA using cell culture supernatants.

### 2.5 Linalool Analysis

GC/MS was performed on a Hewlett-Packard GC/MS system (Agilent G1503A) equipped with a 5,973 Mass Selective Detector and a 59864B Ionization Gauge Controller (Agilent Technologies). A J&W DM-225 GC column (30 m; i.d.: 0.25 mm; film thickness: 0.25 µm; Agilent Technologies) was used. Injector temperature was 260°C. Helium (0.7 ml/min) was used as a carrier gas. Transfer line temperature was 240°C. The following temperature program was applied: 60°C hold for 1 min, increase to 240°C at 10°C/min followed by 5 min at 240°C. EI ionization was in positive ion mode (electron energy: 2040 V; multiple ion detection modus at *m/z* 93, 105, 121, and 161). Data acquisition was performed by MSD ChemStation D.03 software (Agilent Technologies).

### 2.6 Statistical Data Analysis

Statistical data analyses were performed using GraphPad Prism (version 9.2.0 for macOS; GraphPad Software). The Shapiro-Wilk test was used to check for normal distribution. Multiple group comparisons were performed using the Brown-Forsythe and Welch ANOVA tests, followed by the Dunnett’s T3 multiple comparisons posthoc test (with individual variances computed for each comparison). Probability values *p ≤ 0.05 were considered statistically significant. The asterisks represent significant differences from the control group (*p < 0.05, **p < 0.01, ***p < 0.001, and ****p < 0.0001). All results are expressed as means ± SD of at least three independent experiments.

## 3 Results

### 3.1 Protein analysis

**FIGURE 1:**
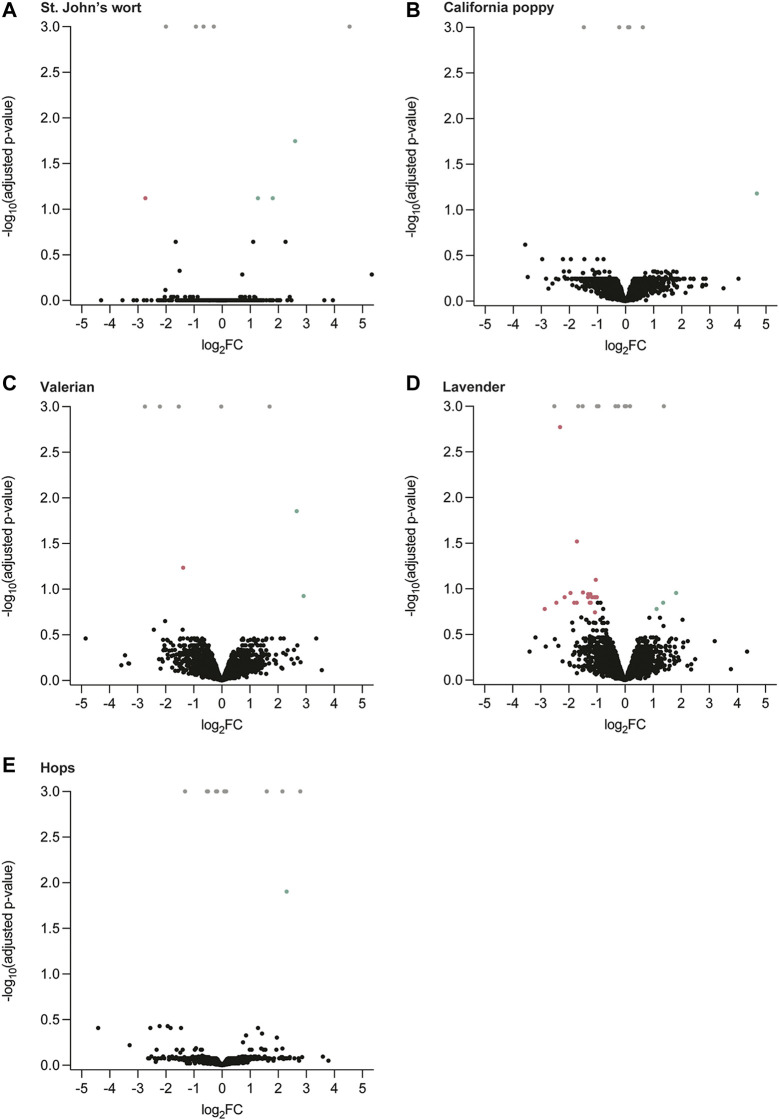
Label-free quantification of proteins of treated (herbal extracts) and untreated (0.06% DMSO) BeWo b30 cells for a period of 48 h. Volcano plots representing identified proteins as log_2_ fold change (FC) ratio of protein intensity (treated/untreated), plotted against the significance as a function of negative log_10_ (adjusted *p*-value). Significantly enriched proteins (adjusted *p*-value ≤ 0.02) are colored accordingly (• log_2_FC > 1, • log_2_FC > −1). Insignificantly different proteins are marked in black. Proteins that were only identified in either the treated or untreated sample are displayed in gray (adjusted *p*-value = 0.001). **(A)** St. John’s wort, **(B)** California poppy, **(C)** valerian, **(D)** lavender, and **(E)** hops.

The influence of hydroalcoholic herbal extracts of St. John’s wort, California poppy, valerian, and hops, as well as lavender essential oil on the proteome of the placental cell line BeWo b30 was assessed (for their phytochemical characterization, see Supporting Information in (Spiess et al., 2021)). Label-free protein quantification showed that none of the five extracts considerably influenced the expression of proteins by up- or downregulation (Figure 1). From approximately 4,000 proteins identified in each case, only a few exceptional proteins were significantly altered after incubation with the herbal extracts of St. John’s wort (4/4,060; RN149, TAP26, MBOA7, and PPR21), California poppy (1/3,353; ANR35), valerian (3/3,423; COPRS, AN32B, and FLOT2), hops (1/3,387; NAGAB), and lavender (24/3,999; CALB2, SPTB1, GEMI, BGLR, UBQL2, RIOX2, STT3B, STT3A, SET, PEBB, SC11A, TTC9C, TBA1B, CCS, CACO2, GALK1, TLE1, TYSY, COQ9, RL36, TF2AA, URM1, S61A1, and EIF3D) were significantly altered (see Supplementary Table S1 for additional information and their functions). Some proteins were only identified in either the treated (herbal extract) or untreated (0.06% DMSO) sample (adjusted *p*-value = 0.001; see Supplementary Table S2–S6 for additional information). Most importantly, no enrichments of biological processes, molecular functions, cellular components, or protein classes and pathways were found when submitting the list of differentially regulated or only in one condition identified proteins to a tool for Gene-Set Enrichment analysis. The amounts of most of the proteins were similar in the absence and presence of the extracts.

### 3.2 Functional Assays

**FIGURE 2:**
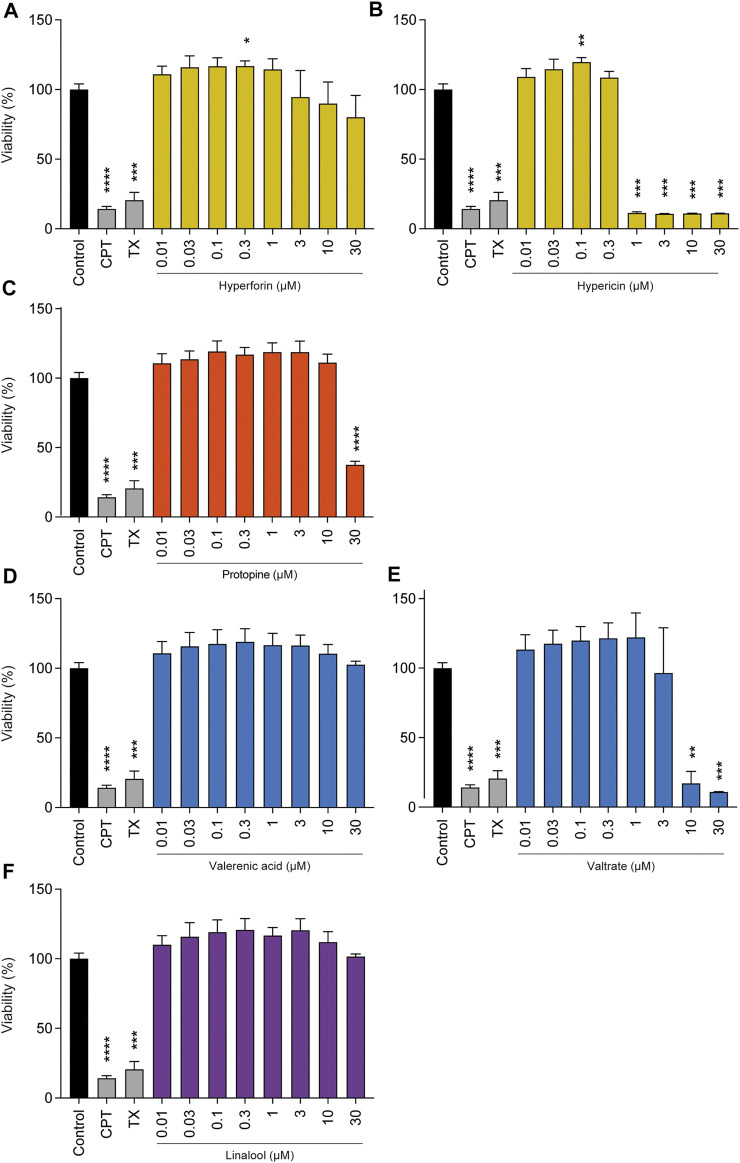
Effects of phytochemicals on cell viability of undifferentiated BeWo b30 cells after 72 h of treatment. Among the phytochemicals present in St. John’s wort, such as hyperforin **(A)** and hypericin **(B)**, only the latter resulted in a significant reduction in cell viability at concentrations of 1, 3, 10, and 30 µM. Protopine **(C)** (present in California poppy) reduced cell viability by 62.4% at a concentration of 30 µM. Of the phytochemicals present in valerian, such as valerenic acid **(D)** and valtrate **(E)**, only the latter resulted in a significant reduction in cell viability at concentrations of 10 and 30 µM. Linalool **(F)** (ingredient of lavender oil) did not show any significant effect in a concentration range from 0.01 up to 30 µM. The effects are shown as fold change compared to the untreated control. Treatments with camptothecin (CPT, 300 µM) and Triton-X-100 (TX, 0.5%) served as toxicity controls. Results were normalized to untreated control signal = 100% (*n* = 3).

To perform an in-depth analysis of the safety of the herbal medicines investigated, phytochemicals that might conceivably cause issues from a safety point of view were chosen. With these individual compounds, we performed various *in vitro* assessments in BeWo cells, starting with effects on cell viability (Figure 2). Hyperforin, which is present in St. John’s wort extract, did not lead to pronounced cytotoxic effects in a concentration range of 0.01–30 µM. However, for hypericin, which is also a major component of the same extract, full viability was only maintained up to a concentration of 0.3 µM, since at 1, 3, 10, and 30 µM it was strongly reduced. Protopine, a representative of California poppy, did not reduce cell viability from a concentration range of 0.01–10 μM; a reduction in cell viability was observed at 30 µM only. Valerenic acid, which is present in valerian extract, showed no effect on cell viability up to high concentrations of 30 µM. In contrast, valtrate (also present in valerian extract) decreased viability at 10 and 30 µM by 83% and 89%, respectively. Linalool (contained in lavender essential oil) showed no cytotoxicity after 72 h of incubation at concentrations of up to 30 µM. Since linalool is highly volatile, we investigated whether (calculated) concentration was retained during diverse experimental conditions at 37°C (Supplementary Table S7). Results show that after 3 h, 60–85% of the original linalool was still present in the cell culture medium, whereas after 48 h, this corresponded to 36–44%.

**FIGURE 3:**
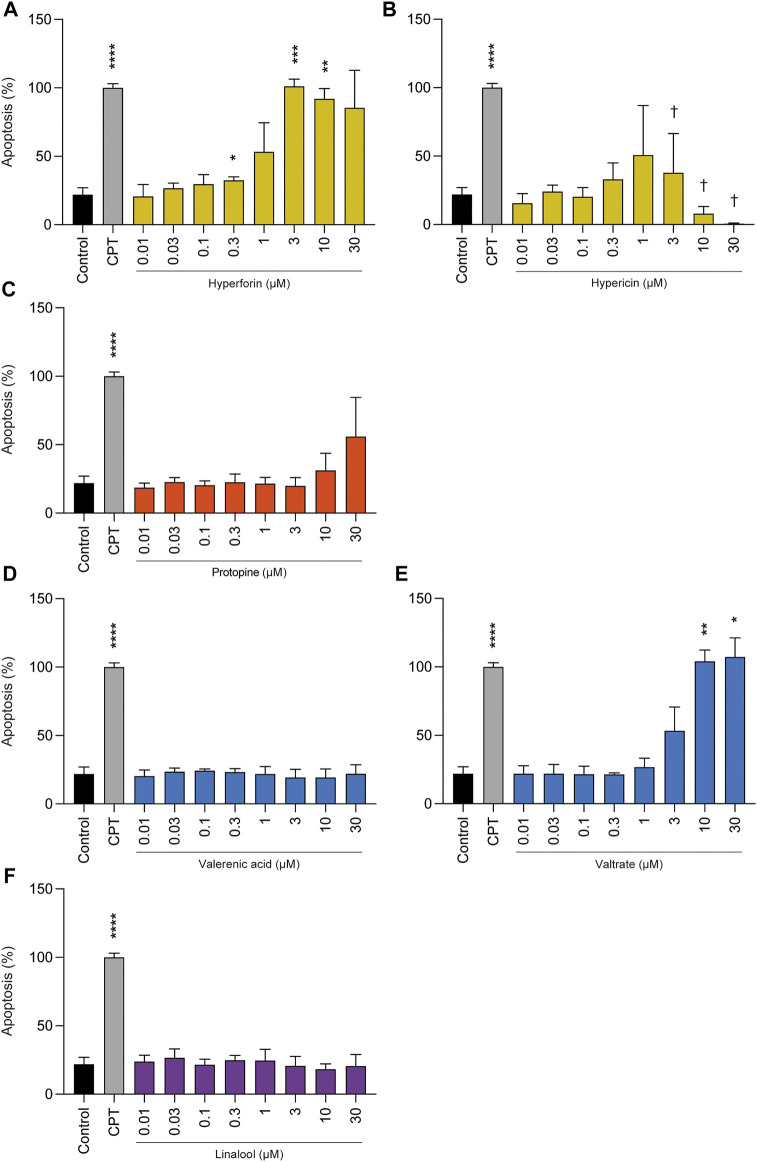
Effects of phytochemicals on cell death of undifferentiated BeWo b30 cells after treatment for 72 h: **(A)** hyperforin, **(B)** hypericin, **(C)** protopine, **(D)** valerenic acid, **(E)** valtrate, and **(F)** linalool. Apoptosis only significantly increased for the highest concentrations of hyperforin (≥3 µM), and valtrate (≥10 µM). Hypericin showed a non-significant increase at 1 µM followed by a significant decrease up to the highest concentration due to an overall decrease in detected cells († cell detection was limited due to advanced degradation). Results were normalized to camptothecin (CPT, 300 µM) = 100% (*n* = 3), which was used as positive control for apoptosis.

Protopine, valerenic acid, and linalool did not induce apoptosis at concentrations ranging from 0.01 to 30 µM (Figure 3). However, hyperforin at concentrations of 3 µM and above, and valtrate at concentrations of 10 and 30 µM revealed increased apoptotic levels compared to control. Hypericin showed a clear-cut even if not significant increase at 1 µM. At higher concentrations, and also in accordance with the results of the viability assay, the number of total detected cells was extremely low (not shown); results on the percentage of apoptotic cells concern only the few detectable cells.

**FIGURE 4:**
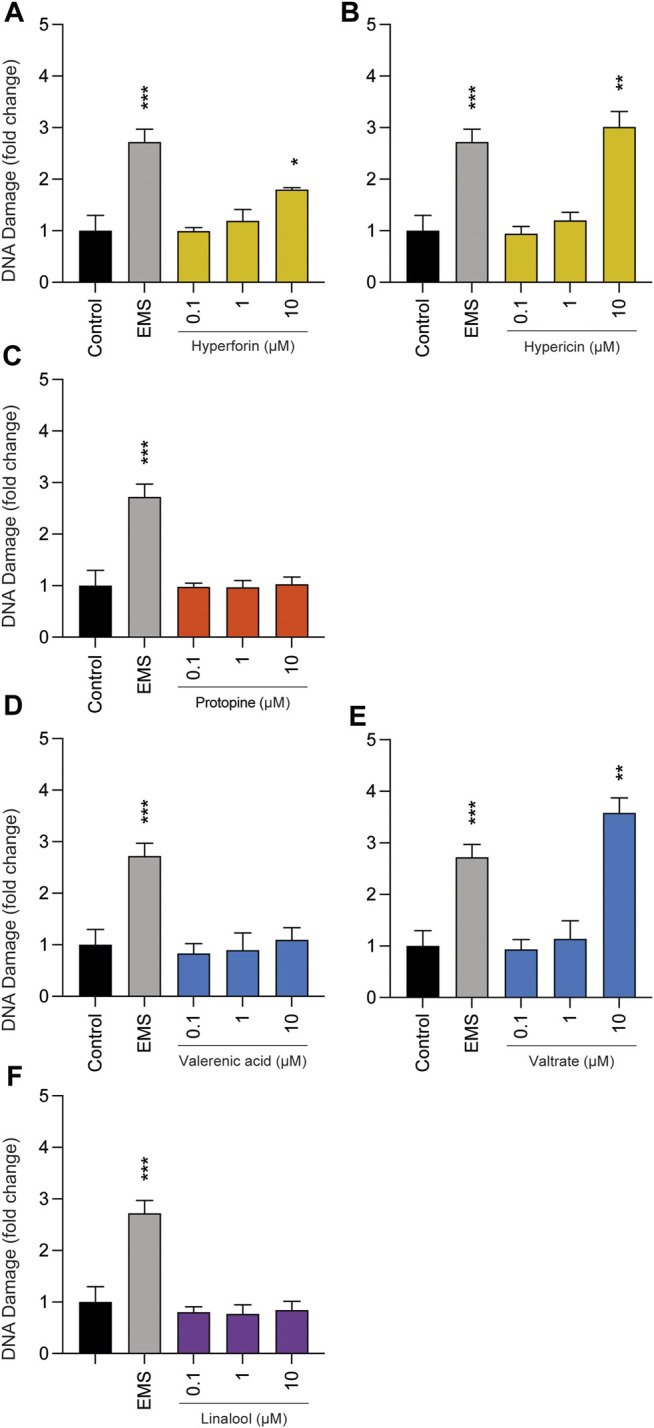
Effects of phytochemicals **(A)** hyperforin, **(B)** hypericin, **(C)** protopine, **(D)** valerenic acid, **(E)** valtrate, and **(F)** linalool on tail DNA in undifferentiated BeWo b30 cells after exposure for 3 h. No significant genotoxic effects were observed at concentrations of 0.1 and 1 µM. Only the highest concentrations (10 µM) of hyperforin, hypericin, and valtrate led to increased DNA damage of BeWo b30 cells. Results were calculated as fold change compared to the untreated control. Ethyl methanesulfonate (EMS, 3 mM) was used as a positive control (*n* = 3).

Comet assays were performed to determine whether the selected phytochemicals can lead to possible genotoxicity at concentrations up to 10 µM (Figure 4). Repetitively, protopine, valerenic acid, and linalool were unremarkable, as predominantly intact nuclear DNA was detected after 3 h of cell treatment; thus none of these substances led to a significant increase in tail DNA. Hyperforin, hypericin, and valtrate showed genotoxicity in this assay, but only when the highest concentration of 10 µM was applied.

**FIGURE 5:**
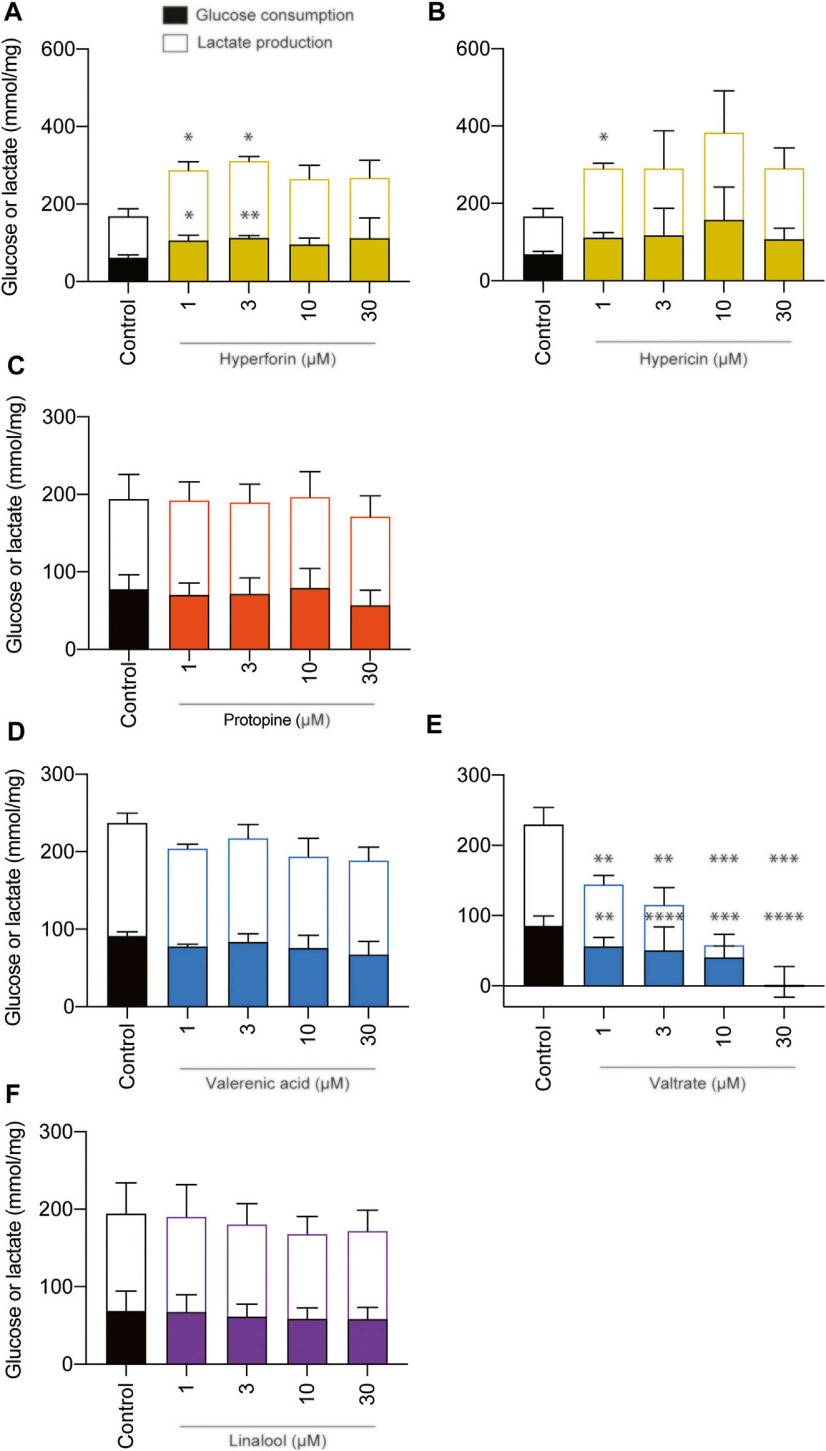
Effects of phytochemicals on glucose consumption and lactate production in undifferentiated BeWo b30 cells after treatment for 48 h. Data were normalized per amount of protein (mg). Control consisted of cell culture media containing 0.2% of DMSO. Data were obtained from three independent experiments (*n* = 3; in triplicate) and are shown as mean ± SD: *p < 0.05. A statistically significant impairment of metabolic activity could not be detected at any of the test concentrations (1, 3, 10, and 30 µM) of the following phytochemicals: protopine **(C)**, valerenic acid **(D)**, and linalool **(F)**. However, valtrate **(E)** decreased the glycolytic metabolism at concentrations of 1, 3, 10, and 30 µM. Phytochemicals of St. John’s wort led to increased glucose consumption and concomitant lactate production in the case of hyperforin treatment **(A)** at 1 and 3 µM and increased lactate concentrations in the case of hypericin **(B)** at 1 µM.

The effects of phytochemicals on metabolic parameters, such as glucose and lactate, were examined and expressed as consumption or production, respectively (Figure 5). Data were normalized to the protein content. Valerian was the only phytochemical which led to changes in glycolytic metabolism at concentrations of 1, 3, 10, and 30 µM by significantly reducing glucose consumption and lactate production of BeWo b30 cells. None of the remaining phytochemicals (hyperforin, hypericin, protopine, valerenic acid, and linalool) affected the metabolic activity of viable BeWo b30 cells when tested at high concentrations of up to 30 µM. Glucose and lactate concentrations of cell supernatants were not statistically different from those of the untreated control. However, hyperforin led to a significant increase in glucose consumption and a concomitant increase in lactate production at lower concentrations of 1 and 3 µM.

**FIGURE 6:**
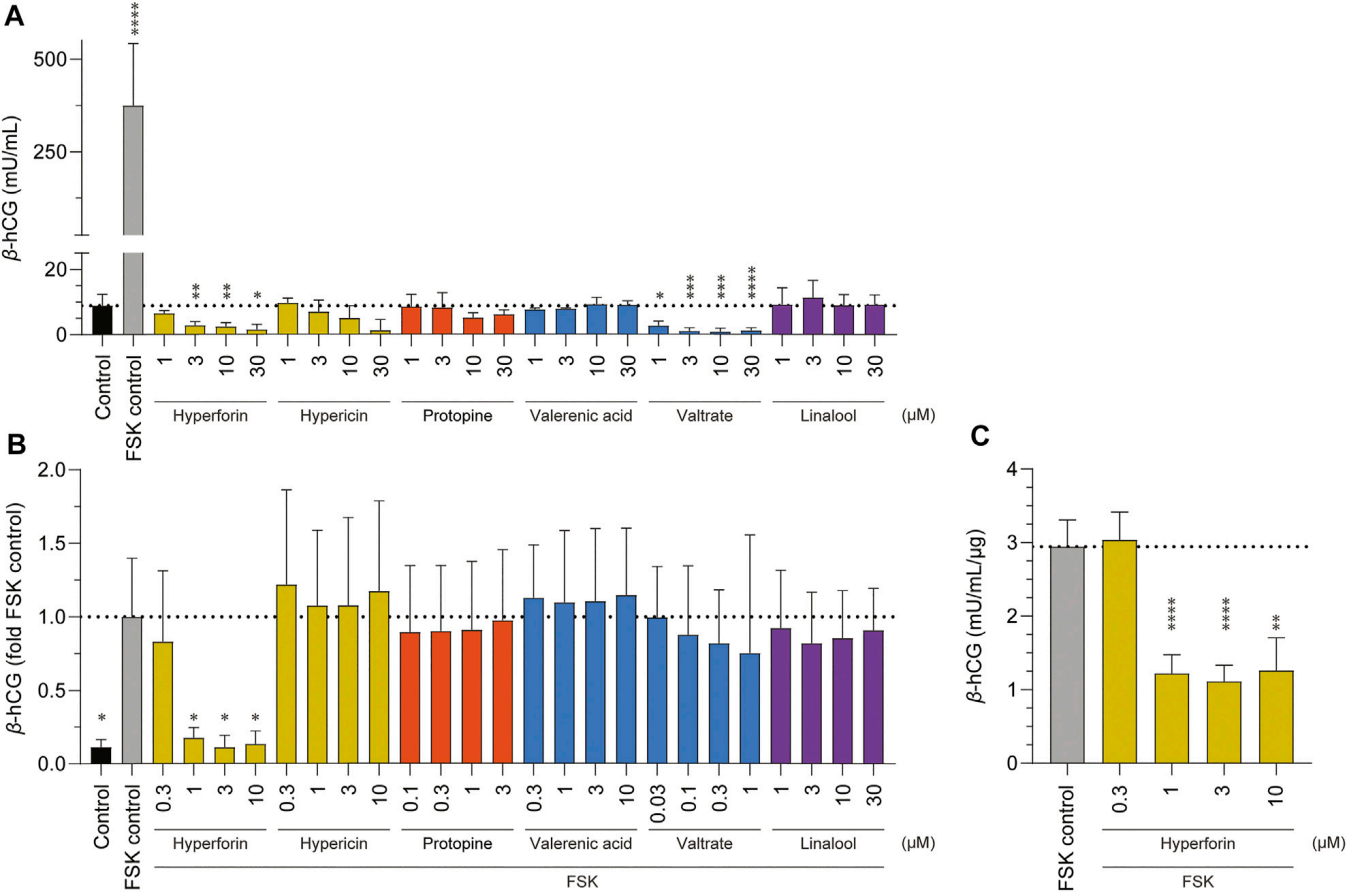
Effects of various phytochemicals on the production of *β-*hCG in BeWo b30 cells. Control consisted of cell culture media containing 0.2% DMSO; 5 µM forskolin (FSK) was used for the FSK control. Data are presented as mean ± SD of at least three independent experiments (*n* = 3–4; in triplicate): *p < 0.05. **(A)** Comparison of *β-*hCG secretion of BeWo b30 cells upon 48-h treatment with increasing concentrations of phytochemicals (1, 3, 10, and 30 µM) vs. FSK control. **(B)** Effects on inhibition of FSK-induced differentiation of BeWo b30 cells. FSK treatment (5 µM) led to increased *β-*hCG levels in all phytochemicals after an incubation of 48 h, except for hyperforin, where the FSK-induced differentiation was inhibited at concentrations of 1, 3, and 10 µM. Based on preliminary data (not shown), different concentration gradients (ranging from 0.03 up to 30 µM) of phytochemicals were individually determined in advance (before exposure). Cells were pre-treated with the different phytochemicals for 24 h, before the addition of FSK for another 24 h. **(C)** Effects of hyperforin on inhibition of FSK-induced differentiation of BeWo b30 cells normalized per amount of protein (μg). FSK treatment (5 µM) led to decreased *β-*hCG levels after an hyperforin incubation of 48 h at concentrations of 1, 3, and 10 µM.

Finally, the impact of the various phytochemicals on the induction of placental cell differentiation was examined, first, by measuring the secretion of the differentiation marker *β-*hCG (Figure 6A), and second, by the opposite approach, namely whether 24 h pre-incubation with the various phytochemicals could inhibit cell differentiation (as induced by FSK; Figures 6B,C). In the first measurement, and after the addition of FSK as a positive control, there was a 42-fold increase in *β-*hCG levels, which is characteristic of BeWo cell differentiation. In contrast, none of the six phytochemicals (hyperforin, hypericin, protopine, valerenic acid, valtrate, and linalool) triggered an increase in *β-*hCG production in BeWo cells. For the inhibition assay of placental cell differentiation, only non-toxic concentrations of each phytochemical (based on preliminary data, not shown) were chosen (Figure 6B). In these assays also, the addition of FSK for 24 h and pre-incubation with cell culture medium for 24 h resulted in 9-fold increased *β-*hCG levels. Under these conditions, most of the phytochemicals (in different concentrations ranging from 0.03 up to 30 µM) had no statistically significant inhibitory effect on the FSK-induced placenta cell differentiation. However, a 24 h pre-incubation with the highest concentrations of hyperforin (1, 3, and 10 µM) did inhibit the following differentiation of BeWo b30 cells. To confirm this, the assay was repeated on a bigger scale to show the hormone concentration normalized to the amount of protein (µg) (Figure 6C). Again, pre-incubation with hypericin (1, 3, and 10 µM) was shown to inhibit FSK-induced differentiation.

## 4 Discussion

### 4.1 Main Findings

None of the hydroalcoholic extracts from St. John’s wort, California poppy, valerian, and hops, nor lavender essential oil significantly affect the protein expression of BeWo b30 cells after 2 days of incubation at a concentration of 30 μg/ml. When we focused on compounds that might conceivably cause safety issues, no decreased cell viability and induction of apoptosis could be observed in a concentration range between 0.01 and 0.3 µM. However, hypericin (≥1 µM), protopine (30 µM) and valtrate (≥10 µM) led to cytotoxic effects and thus decreased the viability of BeWo b30 cells, while hyperforin (≥3 µM), hypericin (≥1 µM), and valtrate (≥10 µM) induced cell apoptosis. No genotoxic effects were observed for any of the tested phytochemical concentrations of 0.1 and 1 µM. Hyperforin, hypericin, and valtrate were only DNA-damaging at elevated concentrations of 10 µM. A 48-h exposure of valtrate (≥1 µM) resulted in reduced glucose consumption and thus reduced lactate production by placental cells; all incubations with the other phytochemicals at concentrations ranging from 1 to 30 µM resulted in viable cells with normal glycolytic metabolism. None of the tested phytochemicals were able to induce or inhibit BeWo b30 cell differentiation, except hyperforin, which was able to inhibit FSK-induced cell differentiation at concentrations of ≥1 µM. To summarize (Table 1), protopine, valerenic acid, and linalool were very inconspicuous in all *in vitro* experiments. Valtrate resulted in cytotoxic, apoptotic, and genotoxic effects (≥10 µM) which were also reflected in reduced metabolic activity (≥1 µM). Hyperforin and hypericin, the two constituents of St. John’s wort, also showed increased toxicity at concentrations ≥1 μM, with the former inhibiting placental cell differentiation (≥1 µM) as well. To put the potential toxicity of the phytochemicals in perspective, available data on achievable plasma concentrations are also presented in Table 1.

**TABLE 1:** Summary of the effects^a^ of plant extracts and their constituents on cytotoxicity, genotoxicity, metabolic activity, and their ability to induce or inhibit cell differentiation, in addition to reported plasma concentrations in literature.

Plant/Constituent	Viability	Apoptosis	Genotoxicity	Metabolic Activity		Differentiation		Reported plasma concentrations
				Glucose	Lactate	Induction	Inhibition
St. John’s wort (µg/ml)	↓ (100)	↑ (100)	↔	↔	↔	↔	↔	
Hyperforin (µM)	↔	↑ (3)	↑ (10)	↔	↔	↔	↑ (1)	0.3 (Agrosi et al., 2000)
Hypericin (µM)	↓ (1)	↑ (1)	↑ (10)	↔	↔	↔	↔	0.0088 (Schulz et al., 2005)
California poppy (µg/ml)	↓ (100)	↔	↔	↔	↔	↔	↔	
Protopine (µM)	↓ (30)	↔	↔	↔	↔	↔	↔	n.d
Valerian (µg/ml)	↓ (100)	↑ (100)	↔	↔	↔	↔	↔	
Valerenic acid (µM)	↔	↔	↔	↔	↔	↔	↔	18.2 (Anderson et al., 2005)
Valtrate (µM)	↓ (10)	↑ (10)	↑ (10)	↓ (1)	↓ (1)	↔	↔	n.d
Lavender (µg/ml)	↔	↔	↔	↔	↔	↔	↔	
Linalool (µM)	↔	↔	↔	↔	↔	↔	↔	0.85 (Müller et al., 2015)
Hops (µg/ml)	↓ (100)	↔	↔	↔	↔	↔	↔	

a Only the lowest concentration that did cause a clear-cut effect is shown; n.d. = not determined.

### 4.2 Strengths and Limitations

A major strength of this *in vitro* study is the LC-MS/MS based proteomics approach, which allowed us to study activation or inhibition of pathways important for cell viability, proliferation, and differentiation. Moreover, and as done before with extracts, conceivably critical phytochemicals were studied using a variety of well-established assays with shorter (3 h) and longer (72 h) exposure times, including a wide range of concentrations (from 0.01 up to 30 µM). Because of the lack or limited applicability of pharmacokinetic data, these concentrations were chosen to cover up to a hundred times the maximum achievable plasma concentrations after oral application (compare with (Agrosi et al., 2000)). One limitation of the relevance of our data to everyday practice is that most manufacturers do not provide information on the contents of the individual natural products, which makes it very difficult to discuss physiologically relevant concentration ranges of single phytochemicals. In addition, we here characterized the *in vitro* safety of a few compounds only, which is another limitation since the herbal medicines are multicomponent mixtures; the reader is, however, referred to our previous work (Spiess et al., 2021) for the results obtained with the corresponding extracts in the same functional assays. Finally, it is important to remember that the cell line represents only one layer of the placenta and thus does not fully reflect the biological environment of the placenta barrier and that ADME aspects are not considered in the used *in vitro* models.

### 4.3 Herbal Medicines and NMD Treatment

In the following, our present observations relevant for a safety assessment of each of the herbal medicines are discussed in the context of available literature.

Incubation with **St. John’s wort** extract did not induce relevant changes in protein expression. Out of 4,060 detected proteins, only 3 were up-regulated (RN149, TAP26, and MBOA7) and 1 was down-regulated (PPR21). Functional assays with two biologically highly active phytochemicals revealed that for **hyperforin**, there is no cause for concern about concentrations ≤0.3 µM, as apoptosis (≥3 µM), genotoxicity (10 µM), and inhibition of differentiation (≥1 µM) were increased only at the indicated concentrations. Concentrations of 1 µM of hyperforin are at least 3 times higher than maximum achieved plasma concentrations upon treatment with common preparations. These vary widely depending on daily doses tested, dosing regimen, formulation (soft/hard gelatin capsules), and manufacturer (Agrosi et al., 2000; Schulz et al., 2005; Vitiello et al., 2005). At high concentrations, hyperforin could pose embryotoxic and teratogenic risks since it inhibited the growth and differentiation of embryonic stem cells and induced apoptosis in fibroblasts (Nakamura et al., 2013). The highest plasma concentration recorded in the literature is 168.35 ng/ml of hyperforin after administration of a soft gelatin capsule (300 mg St. John’s wort dry extract containing 5% hyperforin and 0.3% hypericin, Hammer Pharma SpA), which converts to approximately 0.3 µM. **Hypericin** showed no toxicity up to a concentration of 0.3 µM, as cytotoxicity, apoptosis, and genotoxicity were increased only above 1, 1, and 10 μM, respectively. Hypericin is less abundant in St. John’s wort than hyperforin (Blaschek, 2016), and maximum peak plasma concentrations of 4.43 ng/ml have been reported with multiple dose administration (Schulz et al., 2005), corresponding to approximately 8.8 nM, which is 33 times lower than 0.3 µM. The amount of hypericin as well as hyperforin were recently quantified in different formulations on the Swiss market by HPLC (Schäfer et al., 2019). It was found that the declared and the actually quantified contents differed considerably in some cases. In addition, all formulations contained hypericin, but hyperforin was not detected in two products (Vogel HyperiMed® and Vogel Hyperiforce®) and its amount in other products was very low (Rebalance® and Remotiv®). Since we detected an inhibition of differentiation starting at 1 µM of hyperforin and any alteration in the physiological development of the placenta might be critical from a safety point of view, in our opinion formulations with a low hyperforin content could be preferred in pregnancy. In our previous study on whole extracts, St. John’s wort showed no significant effects up to a concentration of 30 μg/ml extract for all types of *in vitro* experiments, namely cell viability, apoptosis, genotoxicity, metabolic activity, and inhibition/induction of placental cell differentiation (Spiess et al., 2021). In general, there is a lack of adequate clinical studies on the genotoxicity of St. John’s wort, as well as tests on reproductive toxicity, and fertility (Greeson et al., 2001; Avila et al., 2018). Well known issues such as phototoxicity (Onoue et al., 2011) and interactions with other medications (Nicolussi et al., 2020) must be taken care of also during administration in pregnancy. Moreover, a few studies which were either prospective (Kolding et al., 2015) or based on data analysis of a national birth cohort (Moretti et al., 2009) or on claim data (Schäfer et al., 2021) seem to suggest an increased risk of fetal malformation in pregnant women exposed to St. John’s wort preparations. While the results are striking, they should be interpreted with caution.

In the case of **California poppy**, there were no relevant changes in protein expression when BeWo b30 placental cells were incubated with the extract for 2 days; only one protein (ANR35) was significantly upregulated (1/3,353). California poppy, and several other medicinal plants of the Papaveraceae family (e.g., Papaver, Chelidonium, and Argemone, etc.) as well as of other plant families (e.g., Berberidaceae, Fumariaceae, and Ranunculaceae, etc.) (Guinaudeau and Shamma, 1982), contains **protopine**, and this phytochemical was also investigated. We found a minimal cytotoxic potential, as only the viability assay showed slight significance at the highest concentration of 30 µM. To the best of our knowledge, there are no reports of pharmacokinetic studies of protopine in the literature, which makes it difficult to compare the test concentrations we used with physiologically relevant plasma concentrations in humans. Especially ADME studies are needed to predict any *in vivo* effects of protopine. How much protopine is present in commercially available preparations from California poppy is also unknown and requires further clarification. Our previous (Spiess et al., 2021) and current *in vitro* contributions to the study of California Poppy (and protopine) would be in line with a good safety profile.

The proteomics approach is also indicative of good safety for **valerian**. Incubation with the corresponding extract led to no relevant changes in the proteome. In fact, only 2 proteins (COPRS, AN32B) out of 3,423 were significantly upregulated, and 1 protein (FLOT2) was downregulated. In our various experimental set-ups, two biologically active phytochemicals were included. **Valerenic acid** showed neither toxicological effects, nor significant effects on metabolic properties (glucose consumption, lactate production) nor differentiation of BeWo b30 cells in a concentration range between 0.01–30 µM. **Valtrate**, however, showed significant toxic effects, as cytotoxicity, apoptosis, and genotoxicity were increased under the influence of ≥10 µM. In addition, valtrate was also the only ingredient that had significant effects on the metabolic properties of BeWo b30 cells at levels as low as 1 µM. From a translational perspective, these effects must be interpreted with caution, as valepotriates (like valtrate) are very unstable and are easily degraded by exposure to heat, acids or bases (Blaschek, 2016). Also, in the extract used in our previous (Spiess et al., 2021) and present study no valtrate was present. To our knowledge, no data on plasma concentrations of valtrate exist. For valerenic acid, a maximum serum level of 2.8 μg/ml (i.e. 18.2 µM) was reported in one subject after a single administration of 600 mg valerian (Sedonium^TM^, Lichtwer Pharma) via indwelling catheter in the arm vein (Anderson et al., 2005). Upon oral application, lower plasma concentrations are to be expected. As a reminder, we included concentrations of valerenic acid of up to 30 µM in most assays of our study. Since even at these concentrations no effect was observed and, in addition, valerian extract (≥30 μg/ml) had no negative effect on BeWo b30 cells from a toxicological and metabolic point of view, as well as on placental cell differentiation, our work is in line with good safety for valerian in pregnancy. Studies with animals also found that orally administered valerian extract had no adverse effects on fertility or fetal development (Yao et al., 2007).

Finally, **lavender essential oil** did not affect protein expression in any relevant way. Of the 3,999 proteins identified, only 24 were significantly up- (3 proteins) or downregulated (21 proteins). Importantly, no explicit pathway was overly involved. Lavender oil was also highly inconspicuous in all functional assays from our previous study (Spiess et al., 2021). The same assays were now performed with **linalool**, one of the two major components of the essential oil. The results revealed no impairment of BeWo cells at concentrations up to 30 µM initial concentration. Additional experiments showed that some linalool volatizes during the incubations, therefore with time this initial concentration might have been reduced by up to 64% (30 µM would then be reduced to approximately 10 µM). Of particular importance is the comparison with maximum determined plasma concentrations of linalool (Lasea®, Dr. Willmar Schwabe GmbH and Co KG), where peak values of 22 ng/ml (≙ 0.14 µM) and 131 ng/ml (≙ 0.85 µM) were reached after single administration of 80 mg (therapeutic dose) or multiple administrations (14-days application) of 160 mg, respectively (Müller et al., 2015). These concentrations are therefore markedly lower than those used in our study, even when accounting for volatilization. Plasma concentrations upon liniments or embrocations with lavender oil are not known. Regardless of pregnancy, attention should be drawn to the common adverse reactions (<1/10, ≥1/100) such as gastrointestinal disturbances (gastroesophageal reflux, nausea) and allergic skin reactions (urticaria, pruritus, exanthema) (Medicinal product information search platform (AIPS), 2022).

The **hops** extract was very inconspicuous in our proteomics studies, as out of a total of 3,387 proteins only one (NAGAB) was significantly upregulated. Various *in vitro* toxicity assays showed no negative effects either up to 30 μg/ml (cytotoxicity) or even 100 μg/ml (apoptosis, genotoxicity, metabolic activity, and influence on differentiation) (Spiess et al., 2021). To our knowledge there are no concerns about a specific phytochemical from hops. To date, there are no hop cone mono-preparations available in Switzerland. Their dry extracts are often combined with well-established medicinal plants such as valerian root or passionflower herb, as their effects complement each other well (Medicinal product information search platform (AIPS), 2022). Our data imply no significant harm from a toxicological perspective.

## 5 Final Statement

In conclusion, the herbal extracts and some of their constituents of St. John’s wort (hyperforin, hypericin), California poppy (protopine), valerian (valerenic acid, valtrate), lavender (linalool), and hops showed no toxicological abnormalities in a relevant (low) concentration range, suggesting that low doses of these herbal medicines are likely to be safe during pregnancy. Since hyperforin was able to inhibit placental cell differentiation of cytotrophoblasts into syncytiotrophoblasts (≥1 µM), St. John’s wort formulations with a low hyperforin content should preferably be consumed during pregnancy. Further experimental work should focus on potential fetal exposure, evaluating the transplacental transport of hyperforin—and other compounds that might affect relevant cellular processes and/or cause genotoxic effects. Finally and in view of the urgent need for herbal medicines as treatment options for NMDs in pregnancy, more prospective clinical studies should be conducted to evaluate both efficacy and safety of the most promising herbal medicines.

## Data Availability

The datasets presented in this study can be found in online repositories. The names of the repository/repositories and accession number(s) can be found below: https://www.ebi.ac.uk/pride/archive/, PXD031765.

## Abbreviations

*β*-hCG, beta-human chorionic gonadotropin; CPT, camptothecin; EMS, ethyl methanesulfonate; FC, fold change; FSK, forskolin; NMD, non-psychotic mental disorder; TX, Triton-X-100.
