# Supplementary material for: Advanced in Vitro Safety Assessment of Herbal Medicines for the Treatment of Non-Psychotic Mental Disorders in Pregnancy
Source: Front Pharmacol. 2022 Jun 23;13:882997. doi: 10.3389/fphar.2022.882997 (PMC9259859; doi:10.3389/fphar.2022.882997)
Supplement: Supplementary file 1 [file DataSheet1.pdf]

Supporting Information

Advanced *in vitro* safety assessment of herbal medicines for the treatment of non-psychotic mental disorders in pregnancy

Deborah Spiess[1], Moritz Winker[2,3], Alexandra Dolder Behna[1], Carsten Gründemann[2,3], Ana Paula Simões-Wüst[1*]

Affiliations

[1]Department of Obstetrics, University Hospital Zurich, University of Zurich, Zurich, Switzerland

[2]Center for Complementary Medicine, Faculty of Medicine, University of Freiburg, Freiburg, Germany

[3]Translational Complementary Medicine, Department of Pharmaceutical Sciences, University of Basel, Basel, Switzerland

***Correspondence**

PD Dr. Ana Paula Simões-Wüst
University Hospital of Zurich
Department of Obstetrics
Schmelzbergstrasse 12/PF 125, Path G51a
8091 Zurich
Switzerland
Phone: +41 44 255 51 31
Fax: +41 44 255 44 30
anapaula.simoes-wuest@usz.ch

Table 1S. Identified proteins which were significantly influenced (up- or downregulated) after incubations with plant extracts for a period of 48 h in comparison with vehicle only (DMSO treatment).

Plant extract	Gene	Protein	Function (Szklarczyk et al., 2018; The UniProt Consortium, 2019)	log_2FC	p-value	Adjusted p-value
St. John's wort	RN149	E3 ubiquitin-protein ligase	Enables E3 ubiquitin-protein ligase activity	2.60	8.65E-06	0.018
	TAP26	Thyroid transcription factor 1-associated protein 26	Component of the transcription complexes of the pulmonary surfactant-associated protein-B (SFTPB) and -C (SFTPC) which enhances the homeobox protein Nkx-2.1-activated SFTPB and SFTPC promoter activities	1.80	7.52E-05	0.076
	MBOA7	Lysophospholipid acyltransferase 7	Contributes to the regulation of free arachidonic acid in the cell through the remodeling of phospholipids	1.27	7.29E-05	0.076
	PPR21	Protein phosphatase 1 regulatory subunit 21	Regulator of protein phosphatase 1	-2.73	9.15E-05	0.076
California poppy	ANR35	Ankyrin repeat domain-containing protein 35	Unknown	4.68	1.92E-05	0.066
Valerian	COPRS	Coordinator of PRMT5 and differentiation stimulator	Histone-binding protein which plays a role in muscle cell differentiation	2.92	1.02E-04	0.119
	AN32B	Acidic leucine-rich nuclear phosphoprotein 32 family member B	Multifunctional protein which is involved in the regulation of many processes e.g. cell proliferation, apoptosis, cell cycle progression or transcription	2.67	3.98E-06	0.014
	FLOT2	Flotillin-2	Functionally participating in formation of caveolae or caveolae-like vesicles	-1.38	3.31E-05	0.058
Lavender	CALB2	Calretinin	Calcium-binding protein which is abundant in auditory neurons	1.82	1.50E-04	0.111
	SPTB1	Spectrin beta chain, erythrocytic	Major constituent of the cytoskeletal network underlying the erythrocyte plasma membrane	1.36	7.69E-04	0.142
	GEMI	Geminin	Inhibits DNA replication	1.13	1.08E-03	0.166
	BGLR	Beta-glucuronidase	Plays an important role in the degradation of dermatan and keratan sulfates	-1.01	4.24E-04	0.123
	UBQL2	Ubiquilin-2	Plays an important role in the regulation of different protein degradation mechanisms and pathways including ubiquitin-proteasome system (UPS), autophagy and the endoplasmic reticulum-associated protein degradation (ERAD) pathway.	-1.04	5.88E-05	0.080
	RIOX2	Ribosomal oxygenase 2	Leads to an increase in ribosomal RNA expression; may play an important role in cell growth and survival and may be involved in ribosome biogenesis	-1.06	3.70E-04	0.123
	STT3B	Dolichyl-diphosphooligosaccharide-protein glycosyltransferase subunit STT3B	Plays a role in ER-associated degradation (ERAD) pathway that mediates ubiquitin-dependent degradation of misfolded endoplasmic reticulum proteins; required for efficient post-translational glycosylation	-1.07	1.25E-03	0.181

	STT3A	Dolichyl-diphosphooligosaccharide-protein glycosyltransferase subunit STT3A	Similar catalytic subunit with an overlapping function as STT3B	-1.09	4.21E-04	0.123
	SET	Protein SET	Multitasking protein, involved in apoptosis, transcription, nucleosome assembly and histone chaperoning	-1.17	2.74E-04	0.123
	PEBB	Core-binding factor subunit beta	Master-regulates a host of genes specific to hematopoiesis and osteogenesis	-1.22	5.46E-04	0.142
	SC11A	Signal peptidase complex catalytic subunit SEC11A	Component of the microsomal signal peptidase complex which removes signal peptides from nascent proteins as they are translocated into the lumen of the endoplasmic reticulum	-1.23	8.02E-04	0.142
	TTC9C	Tetratricopeptide repeat protein 9C	Protein coding gene; important paralog of the TTC9 gene which has been shown to be hormonally regulated in breast cancer cells and may play a role in cancer cell invasion and metastasis	-1.24	2.23E-04	0.115
	TBA1B	Tubulin alpha-1B chain	Major constituent of microtubules	-1.25	7.26E-04	0.142
	CCS	Copper chaperone for superoxide dismutase	Delivers copper to copper zinc superoxide dismutase	-1.31	2.25E-04	0.115
	CACO2	Calcium-binding and coiled-coil domain-containing protein 2	Required for autophagy-mediated intracellular bacteria degradation	-1.32	3.43E-04	0.123
	GALK1	Galactokinase	Major enzyme for galactose metabolism	-1.50	1.08E-04	0.110
	TLE1	Transducin-like enhancer protein 1	Transcriptional corepressor that binds to a number of transcription factors	-1.71	1.48E-05	0.030
	TYSY	Thymidylate synthase	Contributes to the de novo mitochondrial thymidylate biosynthesis pathway	-1.72	5.96E-04	0.142
	COQ9	Ubiquinone biosynthesis protein COQ9, mitochondrial	Lipid-binding protein involved in the biosynthesis of coenzyme Q, also named ubiquinone, an essential lipid-soluble electron transporter for aerobic cellular respiration	-1.81	5.67E-04	0.142
	RL36	60S ribosomal protein L36	Component of the large ribosomal subunit	-1.94	1.64E-04	0.111
	TF2AA	Transcription initiation factor IIA subunit 1	Component of the transcription machinery of RNA polymerase II and plays an important role in transcriptional activation	-2.15	4.00E-04	0.123
	URM1	Ubiquitin-related modifier 1	Involved in a specific pathway, which is part of tRNA modification	-2.31	4.15E-07	0.002
	S61A1	Protein transport protein Sec61 subunit alpha isoform 1	Plays a crucial role in the insertion of secretory and membrane polypeptides into the endoplasmic reticulum	-2.45	7.85E-04	0.142
	EIF3D	Eukaryotic translation initiation factor 3 subunit D	mRNA cap-binding component of the eukaryotic translation initiation factor 3 (eIF-3) complex, a complex required for several steps in the initiation of protein synthesis of a specialized repertoire of mRNAs	-2.86	1.08E-03	0.166
Hops	NAGAB	Alpha-N-acetylgalactosaminidase	Required for the breakdown of glycolipids	2.30	3.59E-06	0.013

Table 2S. Label-free quantification of proteins which were only identified in either the treated (St. John's wort extract) or untreated (0.06% DMSO) sample.

	Gene	Protein	Function (Szklarczyk et al., 2018; The UniProt Consortium, 2019)
Treated (St. John's wort)	CDIPT	CDP-diacylglycerol-inositol 3-phosphatidyltransferase	Catalyzes the biosynthesis of phosphatidylinositol (PtdIns) as well as PtdIns:inositol exchange reaction
	PPAL	Lysosomal acid phosphatase	Hydrolyzes orthophosphoric monoesters to alcohol and phosphate
	AT12A	Potassium-transporting ATPase alpha chain 2	Catalytic subunit of a H^+/K^+ and/or Na^+/K^+ -ATPase pump which transports K^+ ions (into the cell) in exchange for Na^+ and/or H^+ ions (to the extracellular compartment) across the apical membrane of epithelial cells
Untreated (0.06% DMSO)	TGT	Queuine tRNA-ribosyltransferase catalytic subunit 1	Catalyzes the base-exchange of a guanine (G) residue with queuine (Q) at position 34 (anticodon wobble position) in tRNAs with GU_N anticodons (tRNA-Asp, -Asn, -His and -Tyr), resulting in the hypermodified nucleoside queuosine
	5NT3A	Cytosolic 5'-nucleotidase 3A	Specific activity towards cytidine monophosphate (CMP) and 7-methylguanosine monophosphate (m_7GMP); CMP seems to be the preferred substrate

Table 3S. Label-free quantification of proteins which were only identified in either the treated (valerian extract) or untreated (0.06% DMSO) sample.

	Gene	Protein	Function (Szklarczyk et al., 2018; The UniProt Consortium, 2019)
Treated (valerian)	WIPF1	WAS/WASL-interacting protein family member 1	Plays a role in the reorganization of the actin cytoskeleton; contributes with NCK1 and GRB2 in the recruitment and activation of WASL; may participate in regulating the subcellular localization of WASL, resulting in the disassembly of stress fibers in favor of filopodia formation; plays a role in the formation of cell ruffles
	MBP	Myelin basic protein	Is, with PLP, the most abundant protein component of the myelin membrane in the CNS; formation and stabilization of this compact multilayer arrangement of bilayers
Untreated (0.06% DMSO)	MYL3	Myosin light chain 3	Regulatory light chain of myosin; does not bind calcium
	1B41	HLA class I histocompatibility antigen, B-41 alpha chain	Antigen-presenting major histocompatibility complex class I (MHCI) molecule; in complex with B2M/beta 2 microglobulin displays primarily viral and tumor-derived peptides on antigen-presenting cells for recognition by alpha-beta T cell receptor (TCR) on HLA-B-restricted CD8-positive T cells, guiding antigen-specific T cell immune response to eliminate infected or transformed cells
	CAVN1	Caveolae-associated protein 1	Plays an important role in caveolae formation and organization; essential for the formation of caveolae in all tissues

Table 4S. Label-free quantification of proteins which were only identified in either the treated (hops extract) or untreated (0.06% DMSO) sample.

	Gene	Protein	Function (Szklarczyk et al., 2018; The UniProt Consortium, 2019)
Treated (hops)	NGRN	Neugrin	Plays an essential role in mitochondrial ribosome biogenesis
	ANKZ1	Ankyrin repeat and zinc finger domain-containing protein 1	Plays a role in the cellular response to hydrogen peroxide and in the maintenance of mitochondrial integrity under conditions of cellular stress; involved in the endoplasmic reticulum (ER)-associated degradation (ERAD) pathway
	DPY30	Protein dpy-30 homolog	Involved in the methylation of histone H3 at 'Lys-4', particularly trimethylation, which represents a specific tag for epigenetic transcriptional activation; may also play an indirect or direct role in endosomal transport
	CLAP1	CLIP-associating protein 1	Microtubule plus-end tracking protein that promotes the stabilization of dynamic microtubules; involved in the nucleation of noncentrosomal microtubules originating from the trans-Golgi network (TGN)
	ITA3	Integrin alpha-3	Integrin alpha-3/beta-1 is a receptor for fibronectin, laminin, collagen, epiligrin, thrombospondin and CSPG4
	SP100	Nuclear autoantigen Sp-100	Together with PML, this tumor suppressor is a major constituent of the PML bodies, a subnuclear organelle involved in a large number of physiological processes including cell growth, differentiation and apoptosis; may play a role in angiogenesis, controlling endothelial cell motility and invasion
	LRCH4	Leucine-rich repeat and calponin homology domain-containing protein 4	This gene encodes a protein that contains leucine-rich repeats (LRR) at its amino terminus and that is known to be involved in ligand binding; carboxyl terminus may act as a membrane anchor
	SET1A	Histone-lysine N-methyltransferase SETD1A	Methylates 'Lys-4' of histone H3, when part of the SET1 histone methyltransferase (HMT) complex, but not if the neighboring 'Lys-9' residue is already methylated; H3 'Lys-4' methylation represents a specific tag for epigenetic transcriptional activation; may play a role in synaptic function and the development of neurons
Untreated (0.06% DMSO)	HMCES	Abasic site processing protein HMCES	Acts as an enzyme that recognizes and binds abasic sites in ssDNA at replication forks and chemically modifies the lesion by forming a covalent cross-link with DNA; promotes error-free repair of abasic sites by acting as a 'suicide' enzyme that is degraded, thereby protecting abasic sites from translesion synthesis (TLS) polymerases and endonucleases that are error-prone and would generate mutations and double-strand breaks; acts as a protease: mediates autocatalytic processing of its N-terminal methionine in order to expose the catalytic cysteine
	GPX4	Phospholipid hydroperoxide glutathione peroxidase	Essential antioxidant peroxidase that directly reduces phospholipid hydroperoxide even if they are incorporated in membranes and lipoproteins; plays a key role in protecting cells from oxidative damage by preventing membrane lipid peroxidation
	PRIO	Major prion protein	Unclear primary physiological function; may play a role in neuronal development and synaptic plasticity; may play a role in iron uptake and iron homeostasis

Table 5S. Label-free quantification of proteins which were only identified in either the treated (California Poppy extract) or untreated (0.06% DMSO) sample.

	Gene	Protein	Function (Szklarczyk et al., 2018; The UniProt Consortium, 2019)
Treated (California poppy)	NOL3	Nucleolar protein 3	Isoform 1: May be involved in RNA splicing<br>Isoform 2: Functions as an apoptosis repressor that blocks multiple modes of cell death; inhibits extrinsic apoptotic pathways; inhibits intrinsic apoptotic pathway in response to a wide range of stresses, through its interaction with BAX resulting in BAX inactivation, preventing mitochondrial dysfunction and release of pro-apoptotic factors; Inhibits calcium-mediated cell death by functioning as a cytosolic calcium buffer, dissociating its interaction with CASP8 and maintaining calcium homeostasis; negatively regulates hypoxia-induced apoptosis in part by inhibiting the release of cytochrome c from mitochondria in a caspase-independent manner; inhibits TNF-induced necrosis by preventing TNF-signaling pathway through TNFRSF1A interaction abrogating the recruitment of RIPK1 to complex I; promotes vascular remodeling through inhibition of apoptosis and stimulation of proliferation, in response to hypoxia
Untreated (0.06% DMSO)	NUDT3	Diphosphoinositol polyphosphate phosphohydrolase 1	May play a role in signal transduction; acts as a negative regulator of the ERK1/2 pathway
	GSTM1	Glutathione S-transferase Mu 1	Conjugation of reduced glutathione to a wide number of exogenous and endogenous hydrophobic electrophiles; involved in the formation of glutathione conjugates of prostaglandin A2 (PGA2) and prostaglandin J2 (PGJ2)
	PURB	Transcriptional activator protein Pur-beta	Capacity to bind repeated elements in single-stranded DNA; plays a role in the control of vascular smooth muscle (VSM) alpha-actin gene transcription as repressor in myoblasts and fibroblasts; participates in transcriptional and translational regulation of alpha-MHC expression in cardiac myocytes by binding to the purine-rich negative regulatory (PNR) element
	YJU2	Splicing factor YJU2	Part of the spliceosome which catalyzes two sequential transesterification reactions, first the excision of the non-coding intron from pre-mRNA and then the ligation of the coding exons to form the mature mRNA; may protect cells from TP53-dependent apoptosis upon dsDNA break damage through association with PRP19-CD5L complex
	SPCS1	Signal peptidase complex subunit 1	Component of the microsomal signal peptidase complex which removes signal peptides from nascent proteins as they are translocated into the lumen of the endoplasmic reticulum; plays a key role in the post-translational processing of flaviviral structural proteins prM, E, and NS1

Table 6S. Label-free quantification of proteins which were only identified in either the treated (levender extract) or untreated (0.06% DMSO) sample.

	Gene	Protein	Function (Szklarczyk et al., 2018; The UniProt Consortium, 2019)
Treated (lavender)	AAAS	Aladin	Plays a role in the normal development of the peripheral and central nervous system
	PNKP	Bifunctional polynucleotide phosphatase/kinase	Plays a key role in the repair of DNA damage, functioning as part of both the non-homologous end-joining (NHEJ) and base excision repair (BER) pathways; through its two catalytic activities, PNK ensures that DNA termini are compatible with extension and ligation by either removing 3'-phosphates from, or by phosphorylating 5'-hydroxyl groups on, the ribose sugar of the DNA backbone
	REX1B	Required for excision 1-B domain-containing protein	Uncharacterized protein
	FHL3	Four and a half LIM domains protein 3	Recruited by SOX15 to FOXK1 promoters where it acts as a transcriptional coactivator of FOXK1
	CO4A5	Collagen alpha-5(IV) chain	Type IV collagen is the major structural component of glomerular basement membranes (GBM), forming a 'chicken-wire' meshwork together with laminins, proteoglycans and entactin/nidogen
	SEM7A	Semaphorin-7A	Plays an important role in integrin-mediated signaling and functions both in regulating cell migration and immune responses. Promotes formation of focal adhesion complexes, activation of the protein kinase PTK2/FAK1 and subsequent phosphorylation of MAPK1 and MAPK3; promotes production of proinflammatory cytokines by monocytes and macrophages; plays an important role in modulating inflammation and T-cell-mediated immune responses; promotes axon growth in the embryonic olfactory bulb
	PJA2	E3 ubiquitin-protein ligase Praja-2	Responsible for ubiquitination of cAMP-dependent protein kinase type I and type II-alpha/beta regulatory subunits and for targeting them for proteasomal degradation; essential for PKA-mediated long-term memory processes
	MGST3	Microsomal glutathione S-transferase 3	Catalyzes the oxydation of hydroxy-fatty acids; catalyzes the conjugation of a reduced glutathione to leukotriene A4 in vitro; may participate in the lipid metabolism
	FBLL1	rRNA/tRNA 2'-O-methyltransferase fibrillarin-like protein 1	S-adenosyl-L-methionine-dependent methyltransferase that has the ability to methylate both RNAs and proteins; involved in pre-rRNA processing by catalyzing the site-specific 2'-hydroxyl methylation of ribose moieties in pre-ribosomal RNA; also acts as a protein methyltransferase by mediating methylation of glutamine residues
Untreated (0.06% DMSO)	DC1I1	Cytoplasmic dynein 1 intermediate chain 1	Acts as one of several non-catalytic accessory components of the cytoplasmic dynein 1 complex that are thought to be involved in linking dynein to cargos and to adapter proteins that regulate dynein function; cytoplasmic dynein 1 acts as a motor for the intracellular retrograde motility of vesicles and organelles along microtubules; may play a role in mediating the interaction of cytoplasmic dynein with membranous organelles and kinetochores
	RASF8	Ras association domain-containing protein 8	Essential for maintaining adherens junction function in epithelial cells and has a role in epithelial cell migration.

Table 7S. Relative concentrations of linalool present in cell culture medium at different positions of a 96-well or 24-well plate after 3 h or 24 h, respectively.

Plate	Time (h)	Position	Well	Area (GC)	Linalool (%)*	Mean (%)
96-well	0	Stock	-	21395		100
				22767		
				23020		
	3	Center	D5	18468	86	85
				18293	80	
				20494	89	
		Border	A5	12865	60	60
				14684	64	
				12946	56	
24-well	0	Stock	-	410938		100
				381251		
				369752		
	48	Center	B4	165974	40	44
				172929	45	
				167115	45	
			C4	153577	37	41
				159509	42	
				163896	44	
		Border	A1	142483	35	36
				117050	31	
				152353	41	

* Calculated as (area t_{24})/(area stock t_0)*100

References

Szklarczyk, D., Gable, A.L., Lyon, D., Junge, A., Wyder, S., Huerta-Cepas, J., et al. (2018). STRING v11: protein–protein association networks with increased coverage, supporting functional discovery in genome-wide experimental datasets. *Nucleic Acids Res.* 47(D1), D607-D613. doi: 10.1093/nar/gky1131.

The Uniprot Consortium (2019). UniProt: a worldwide hub of protein knowledge. *Nucleic Acids Res.* 47(D1), D506-D515. doi: 10.1093/nar/gky1049.
